# Biochemistry of Bacterial Biofilm: Insights into Antibiotic Resistance Mechanisms and Therapeutic Intervention

**DOI:** 10.3390/life15010049

**Published:** 2025-01-02

**Authors:** Kashish Azeem, Sadaf Fatima, Asghar Ali, Ayesha Ubaid, Fohad Mabood Husain, Mohammad Abid

**Affiliations:** 1 Medicinal Chemistry Laboratory, Department of Biosciences, Jamia Millia Islamia, Jamia Nagar, New Delhi 110025, India; rs.kazeem@jmi.ac.in (K.A.); sadaffatima141@gmail.com (S.F.); asgharali@jamiahamdard.ac.in (A.A.); ashislam98@gmail.com (A.U.); 2 Clinical Biochemistry Laboratory, Department of Biochemistry, School of Chemical and Life Science, Jamia Hamdard, New Delhi 110062, India; 3 Department of Food Science and Nutrition, King Saud University, Riyadh 11451, Saudi Arabia

**Keywords:** biofilms, antimicrobial resistance, quorum-sensing inhibitor, nanomaterials, immunomodulators

## Abstract

Biofilms, composed of structured communities of bacteria embedded in a self-produced extracellular matrix, pose a significant challenge due to their heightened resistance to antibiotics and immune responses. This review highlights the mechanisms underpinning antibiotic resistance within bacterial biofilms, elucidating the adaptive strategies employed by microorganisms to withstand conventional antimicrobial agents. This encompasses the role of the extracellular matrix, altered gene expression, and the formation of persister cells, contributing to the recalcitrance of biofilms to eradication. A comprehensive understanding of these resistance mechanisms provides a for exploring innovative therapeutic interventions. This study explores promising avenues for future research, emphasizing the necessity of uncovering the specific genetic and phenotypic adaptations occurring within biofilms. The identification of vulnerabilities in biofilm architecture and the elucidation of key biofilm-specific targets emerge as crucial focal points for the development of targeted therapeutic strategies. In addressing the limitations of traditional antibiotics, this review discusses innovative therapeutic approaches. Nanomaterials with inherent antimicrobial properties, quorum-sensing inhibitors disrupting bacterial communication, and bacteriophages as biofilm-specific viral agents are highlighted as potential alternatives. The exploration of combination therapies, involving antimicrobial agents, biofilm-disrupting enzymes, and immunomodulators, is emphasized to enhance the efficacy of existing treatments and overcome biofilm resilience.

## 1. Introduction

The idea of biofilm microbial populations forming on extracellular matrix-encased surfaces has a long history that was first observed by Anton Van Leeuwenhoek in the 17th century, who first saw microbial aggregates in dental plaques scraped from his teeth [1]. Additionally, Bill Costerton’s groundbreaking research in the 1970s marked the start of the modern era of biofilm research. The advancement of confocal laser scanning microscopy (CLSM) in the 1980s and 1990s made it possible to visualize biofilms in detail, exposing their intricate three-dimensional structure and dynamic properties [2]. Molecular biology techniques were applied in the 1990s and early 2000s, and at the same time, important genes and regulatory pathways, including quorum-sensing mechanisms that are essential for the production of biofilms, were discovered [3]. By the late 20th century, biofilms had been known for their role in chronic infections, especially in medical settings. The understanding of bacterial life was dramatically altered by Costerton’s study, highlighting the prevalence and significance of biofilms in industrial, clinical, and ecological environments. Certain genes govern the synthesis of chemicals required for microbial attachment to surfaces and the subsequent growth of biofilms, which in turn controls the formation of biofilms. These genes are activated when bacteria encounter favorable conditions for biofilm creation, resulting in complex microbial communities forming on surfaces [4]. This control encompasses the synthesis of essential elements such as alginate, mechanisms for stress response, and additional components that support the growth and stability of the biofilm. The intricate process by which bacteria transition from planktonic to sessile lifestyles and adapt to live in a biofilm on a solid surface is demonstrated by the differential expression of several genes [5,6]. Phosphomannomutase, encoded by the *algC* gene, is crucial for alginate synthesis, a key biofilm matrix component. Other genes, such as *algD*, *algU*, and *rpoS*, contribute to biofilm formation by regulating alginate production and stress responses. Additionally, about 45 genes exhibit variations in expression between sessile and planktonic bacteria. A biofilm is a colony of bacteria that are tightly bound to one another or a surface and are encased in an extracellular polymeric matrix that provides protection [7,8]. The EPS is composed of polysaccharides, extracellular proteins, lipids, nucleic acids (e-DNA and e-RNA), and secondary metabolites [9,10]. This matrix serves to bind the cells closely together, enabling the exchange of genetic material, facilitating quorum-sensing, and helping the colonies adhere to the surfaces. Bacteria form biofilms that may comprise a single microbial species or multiple microbial species on a diverse range of surfaces, both biotic and abiotic [4]. These include living tissues in plants, animals, and humans, as well as non-living objects like glass surfaces, metals, plastic products, and medical equipment [11,12]. In cases of infections on medical implants, the biofilm is often dominated by a single pathogen. In plants, biofilm formation can cause plant diseases and impact agricultural and ecological systems [13]. In humans, biofilm-related infections can occur on various body surfaces, leading to dental issues, implant-associated infections, and other health problems. Biofilm-related diseases are often challenging to treat and require specialized approaches in both clinical and environmental settings [14,15]. Bacterial biofilms adhere to a wide range of surfaces, including medical implants and devices like pacemakers, vascular grafts, heart valves, IUDs, prosthetic joints, catheters, sutures, and contact lenses; hence, investigating biofilm formation and control strategies is crucial for improving the design and functionality of medical devices [16]. Biofilm formation initiates the disease process through various mechanisms, such as the breakdown of individual bacterial cells or clusters, the generation of endotoxins, enhanced evasion from host immune system surveillance, and the development of a protective barrier that fosters the emergence of immune-resistant organisms [15]. *Streptococcus mutans* is the primary pathogen in dental caries due to biofilm formation and acid production [17]. *Enterococcus* species, particularly VRE, cause UTIs, endocarditis, and intra-abdominal infections [18]. *Klebsiella pneumoniae* is associated with severe hospital-acquired infections like pneumonia and sepsis, often exacerbated by antibiotic resistance [19]. Biofilm-based infections present a significant challenge in clinical settings due to the increased tolerance of biofilm cells to antimicrobial substances, heavy metals, and toxic chemicals [2,20,21]. Intricate biofilm mechanisms enhance resistance, highlighting unique biofilm features that reduce susceptibility to biocides [22]. Due to their multidrug resistance, ability to evade host defense mechanisms, persistence, and capacity to thrive in various harsh environments, biofilms pose significant health challenges [23]. Investigations into biofilms have catalyzed the creation of novel therapeutic strategies, encompassing biofilm-disrupting enzymes, anti-biofilm coatings for medical devices, and alternative treatments such as bacteriophages and nanoparticles [24]. These innovations aim to enhance the effectiveness of treatment and reduce the burden of biofilm-related infections. The development of novel strategies and special approaches to counter these resilient microbial communities, along with a deeper understanding of their biology, and underlying biofilm-related infections, is essential for the prevention and treatment of biofilm-associated diseases [24,25].

## 2. Biofilm Formation and Growth

Biofilm formation is a complex process that is crucial for the survival of microorganisms in various environments. It involves the production and stabilization of biofilms through cell-to-surface and cell-to-cell interactions, mediated by bacterial polysaccharides. Polysaccharides are essential for initial attachment, quorum-sensing communication, extracellular matrix formation, and overall biofilm stability, making them resistant to disruption and environmental challenges [26]. Biofilms exhibit viscoelastic behavior, allowing them to adapt to surface contours and withstand mechanical stresses [27]. The initial attachment phase involves the recognition and interaction of bacterial appendages or physical forces with surfaces [28]. Adhesion and aggregation follow, establishing an irreversible connection between microorganisms and the surface. Micro-colony formation occurs, involving multiplication and cell division, leading to the establishment of microcolonies with diverse micro-communities [28]. Maturation is the fourth stage, where biofilm-specific genes are expressed, and biofilm matures through signaling molecules and the production of EPS. The final stage is dispersion, where biofilms release planktonic cells due to nutrient scarcity and accumulate toxic metabolic wastes, facilitating the colonization of new surfaces [29].

**Figure 1:**
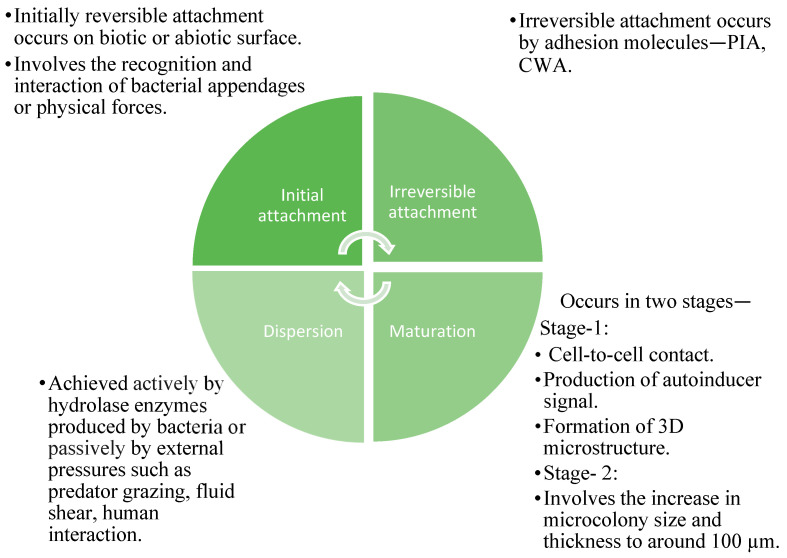
Developmental stages of biofilm formation (PIA—Polysaccharide Intercellular Adhesin, CWA—Cell wall-anchored).

The characteristics of biofilms differ at distinct stages, as illustrated in Figure 1. Throughout these stages, various factors including environmental conditions, bacterial species, and strain types contribute to the unique traits of biofilms.

Biofilm formation involves intricate signaling pathways comprising molecular interactions that govern its different stages, from initiation and maturation to dispersal. A summary of the various molecules, their types, and their role in bacterial biofilm formation are mentioned in Table 1.

### 2.1. Environmental Factors Affecting Biofilm Formation

Bacterial characteristics such as surface charge and physicochemical characteristics like roughness, hydrophobicity, and surface free energy largely influence the early phases of biofilm formation by promoting bacterial adherence [30]. On the other hand, hydrodynamics, temperature, pH, availability of nutrients, and oxygen concentrations are more important environmental variables that affect the long-term development and stability of biofilms [31]. Initial colonization is primarily driven by physicochemical traits, but over time, environmental factors have a bigger influence on the creation and dynamics of biofilms [32].

#### 2.1.1. Hydrodynamics

Different hydrodynamic settings that biofilms are exposed to can affect the matrix of the biofilm. Changes in the availability of nutrients and oxygen, as well as the application of shear pressures, can substantially influence the adhesion of cells to the surface and affect these processes under different environmental situations [33]. These conditions, combined with fluid hydrodynamics, alter the rate at which nutrients, oxygen, and bacterial cells are transported from the medium to the biofilm. In addition to affecting physical characteristics like density and strength, this fluid flow may also regulate the diffusion of nutrients and signaling chemicals throughout the biofilm [33]. In *Pseudomonas aeruginosa*, quorum sensing is influenced by hydrodynamics, as shear stress causes the detachment of cells due to increased flow velocity [34].

#### 2.1.2. Environmental pH

The initial adhesion stage of biofilm formation is highly influenced by the pH of the surrounding environment [35]. Enzymatic activity is sensitive to specific pH conditions, with each enzyme functioning best within its optimal pH range. The ideal pH for polysaccharide secretion differs between species but typically centers around pH 7 for the majority of bacterial strains. Exopolysaccharide synthesis within biofilms functions as a protective strategy against challenging environmental conditions, including fluctuations in pH [35]. Bacteria embedded in biofilms display heightened resistance to pH shifts when compared to planktonic cells. In strongly acidic environments, the gel-like consistency of the biofilm matrix may hinder the swift movement of ions, resulting in a pH gradient forming within the extracellular matrix. Conversely, in alkaline conditions, biofilms often appear disorganized and extremely thin [36].

#### 2.1.3. Temperature

Optimal temperature conditions are key to promoting bacterial development. Elevated temperatures enhance bacterial growth and are associated with increased nutrient uptake. Enzymes, which govern numerous physiological and biochemical processes in bacteria, particularly those involved in nutrient metabolism, are highly temperature sensitive [34]. On the other hand, temperatures below the optimum range can suppress the growth of bacteria due to a slower metabolism rate. Beyond enzymatic activity, ambient temperature also alters the chemical composition of molecules within the cell and around bacteria. For example, bacteria like *S. aureus* demonstrate increased hydrophobicity and stronger attachment to surfaces when cultured at temperatures between 20 and 37 °C [35]. Additionally, temperature affects the presence of surface structures such as flagella, pili, and fimbriae on bacteria, which are crucial for adhesion. For example, a reduction in temperature decreases the adherence ability of aquatic *Pseudomonas* species to the surface, largely due to a decrease in bacterial surface polymer molecules [37]. The characteristics of bacterial exopolysaccharides (EPS), including their viscosity, are also affected by temperature. As the temperature increases, the gel-like matrix of EPS progressively strengthens, shifting into a more fluid state. Therefore, the consistency of polysaccharides tends to stabilize at lower temperatures, which may enhance the potential for bacterial biofilm attachment. Conversely, in some microorganisms, higher temperatures have been shown to strengthen biofilm adhesion to surfaces [36].

#### 2.1.4. Oxygen Availability

The presence of oxygen in the surrounding environment is a key factor shaping biofilm development and structure. Reduced oxygen availability often initiates active dispersal due to decreased metabolic rate, a critical phase in the biofilm life cycle. In certain cases, bacteria located at the base of a biofilm experience lower oxygen levels compared to those near the surface, promoting detachment from the deeper biofilm layers. The phenomenon of sloughing, or the shedding of biofilm layers, has been observed in oxygen-limited environments. The deprivation of oxygen can serve as a detachment cue in some bacteria, such as *E. coli*, highlighting the importance of oxygen availability in biofilm development [36]. In other bacteria, such as *P. aeruginosa*, biofilm formation has been observed under anaerobic growth conditions [38].

#### 2.1.5. Nutrient Levels

Nutrient levels significantly influence the transition from planktonic bacteria to a sessile form, determining whether bacteria will form biofilms or remain free-floating based on the surrounding nutritional conditions [36]. Increased nutrient levels have been shown to enhance biofilm cell densities, such as in drinking water distribution systems [39]. In paper mill water streams, the formation of *Pseudomonas putida* biofilms is accelerated both in rate and quantity under elevated nutrient conditions. Studies indicate that adding glucose as a carbon source to the growth medium promotes faster biofilm development in bacteria like *E. coli* and *P. putida* [40].

### 2.2. Signaling Pathways in Biofilm

The development of biofilms is a highly coordinated process regulated by complex signaling pathways. These pathways enable microorganisms to perceive their surroundings, communicate with one another, and adapt their behavior to enhance biofilm development and sustainability [4].

#### 2.2.1. Quorum Sensing (QS)

N-acyl homoserine lactones (AHLs) exhibit diversity by variation in the length and functionalization of the acyl side chain [41]. A small quantity of autoinducer is extracellularly present during low cell density but it is too diluted to be detected. As cell density rises, autoinducer concentrations exceed a crucial threshold, which triggers autoinducers to bind to the regulatory protein (receptor) complex and subsequently alter target gene expression [42]. Numerous physiological processes, like virulence factor secretion, biofilm formation, antibiotic resistance, and others, are regulated by quorum sensing (QS) (Table 2).

#### 2.2.2. Cyclic-di-GMPHI

**Figure 2:**
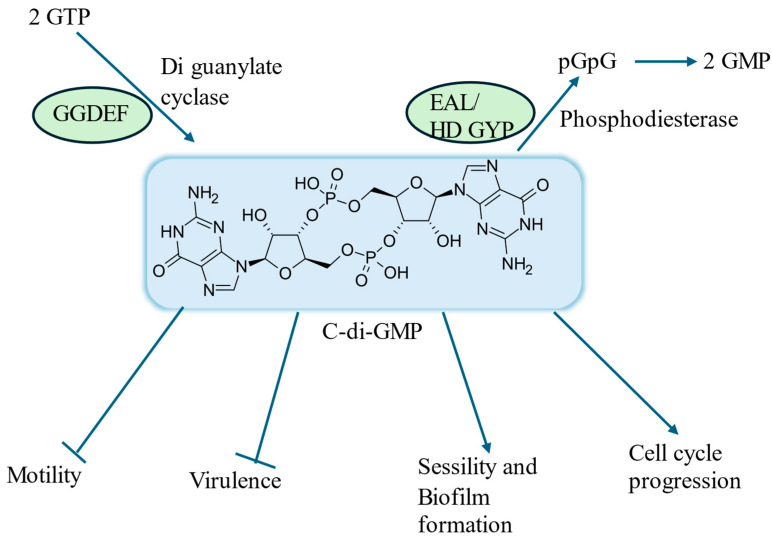
C-di-GMP action in biofilm formation.

Within cellular processes, the secondary messenger c-di-GMP is synthesized via the cyclization of two guanosine triphosphate (GTP) molecules facilitated by diguanylate cyclases (DGCs) harboring GGDEF domains, which possess highly conserved active site residues pivotal for enzymatic catalysis [47]. Concurrently, c-di-GMP undergoes degradation pathways mediated by phosphodiesterases (PDEs) equipped with either EAL or HD-GYP domains. EAL domain-containing PDEs hydrolyze c-di-GMP to pGpG, subsequently leading to the production of GMP, while HD-GYP domain-containing enzymes directly catalyze the conversion of c-di-GMP into GMP [48]. These enzymatic mechanisms serve as fundamental regulators in bacterial cellular signaling and function. Together, these enzymes regulate intracellular c-di-GMP levels (Figure 2).

In most bacteria, effector proteins binding to c-di-GMP regulate the switch from a motile to a sessile lifestyle, leading to diverse outcomes through mechanisms like cell-surface protein localization, protein interactions, transcriptional activation, and DNA binding. The functional role of cyclic di-GMP (c-di-GMP) in various bacterial species is regulated by specific enzymes, as summarized in Table 3.

#### 2.2.3. Small Noncoding RNAs (sRNAs)

The transition between planktonic and biofilm forms is regulated by small noncoding RNAs (sRNAs), essential components of bacterial regulatory networks. The discovery of the RNA-binding protein CsrA and small noncoding RNA CsrB by Romeo and colleagues, which are crucial for *E. coli* biofilm formation and dispersal, led to the identification of many additional sRNAs involved in biofilm regulation in various bacteria. CsrA was the first identified global regulator of biofilm formation [53]. CsrB and CsrC are two sRNAs that inhibit the ability of CsrA to bind target mRNAs. CsrA suppresses mRNA translation in the absence of these sRNAs, increasing cell motility. However, CsrA is sequestered by CsrB and CsrC sRNAs, which permits mRNA translation and encourages the creation of bacterial biofilms [53].

**Figure 3:**
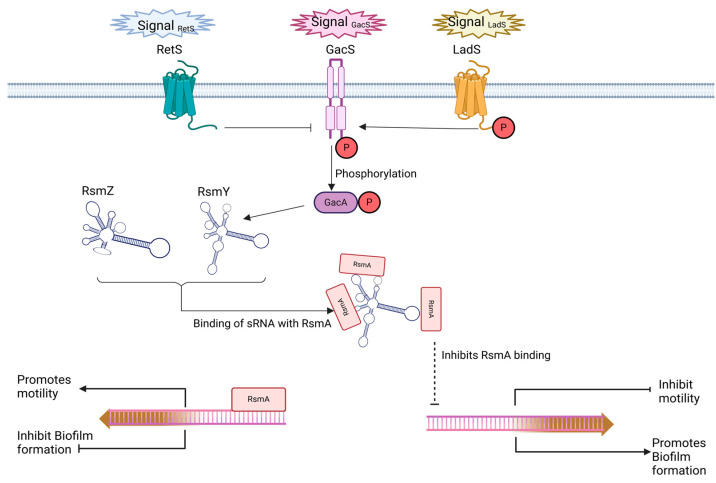
The GAC signal-transduction in *P. aeruginosa*.

The GAC signal-transduction in *P. aeruginosa* regulates biofilm formation. GacS phosphorylates GacA, a process enhanced by LadS and inhibited by RetS. Phosphorylated GacA induces the production of sRNAs (RsmY and RsmZ), which binds with RsmA, a translational repressor that promotes motility and inhibits biofilm formation, thereby facilitating biofilm development (Figure 3) [54]. CsgD is another major biofilm regulator in *E. coli* and *S. typhimurium*, functioning at the transcriptional level. It selectively activates genes for curli fimbriae and extracellular polysaccharides while suppressing those for flagella [55,56]. The production of several sRNAs, in response to different environmental circumstances, such as McaS, RprA, OmrA/OmrB, and GcvB, are involved in the regulation of CsgD activity.

EPS production is regulated by both cyclic di-GMP and quorum-sensing pathways. These pathways coordinate the synthesis of matrix components such as alginate, pel, and psl polysaccharides in species like *P. aeruginosa* [46,57]. Signaling pathway interactions allow biofilms to adapt to changes such as nutrient availability, pH shifts, and oxidative stress.

## 3. Mechanism of Antibiotic Resistance

Biofilms exhibit significantly enhanced resistance to antibiotics and host immune defenses [2]. Bacteria within biofilms exhibit increased antibiotic resistance, which ranges from ten to a thousand fold higher resistance when compared to their planktonic form [58]. When experimented, *Staphylococcus epidermis* in its planktonic stage was reported to be sensitive to vancomycin, while approximately 75% of the isolated strains from a biofilm were resistant to the same antibiotics. Several reasons contribute to increased drug resistance of bacteria within biofilms compared to their planktonic counterparts.

### 3.1. Reduced Penetration of Antibiotics

Biofilms develop an extracellular matrix that serves as a physical barrier, preventing antibiotics and other medications from penetrating the biofilm and reaching the bacteria within. This barrier prevents bacteria from being destroyed, resulting in chronic, recurring infections despite antibiotic treatment. Infrared spectroscopy has demonstrated that the rate of transport of the antibiotic ciprofloxacin to a colonized surface is slower when compared with sterile surfaces [59]. *P. aeruginosa* biofilms overproduce the enzyme β-lactamase in the matrix, which can impair the function and activity of antibiotics before they reach the bacterial cells or reduce the likelihood of β-lactams’ contact with bacteria cells. This limits the exposure of bacteria within the biofilm to sublethal concentrations of antibiotics, allowing them to adapt and develop resistance [60]. The delivery and retention of enzymes that may break down and degrade antibiotics in the extracellular matrix is another crucial function of this diffusion barrier.

### 3.2. Stratified Metabolic Rates Within the Biofilm

Nutrient gradients often occur in biofilms, with the outer layers absorbing more nutrients than the inner layers. This gradient can cause stratification in bacterial metabolic activity [61]. The restricted availability of nutrients influences the nature of the barrier and modifies the bacterial cell membrane, leading to a slow rate of biofilm growth and causing cells to enter a starved state [62]. Bacteria adapt to a state of lower metabolic activity in less nourished areas, making them less sensitive to antibiotics that target actively dividing cells. The difference in the concentration gradients of nutrients, waste products, and signaling molecules leads to heterogeneity within the biofilm.

### 3.3. Quorum Sensing and Cell Density

Bacteria within biofilms can communicate through quorum sensing, (QS) allowing them to coordinate their activities, which can lead to the upregulation of certain genes associated with drug resistance. Once activated, the QS gene initiates the development of the biofilm and subsequently coordinates its maturation and disintegration. The number of bacteria present in a given volume can be ascertained by measuring the amount of AI-signaling molecules secreted by the bacteria in a colony [63]. Although there are many types of QS mechanisms in bacteria, two of the most prevalent depend on oligopeptides and acyl homoserine lactones (AHLs) in Gram-positive and Gram-negative bacteria, respectively. Another type of QS molecule present in both Gram-positive and Gram-negative bacteria uses a family of related molecules known as autoinducer-2 (AI-2). In *P. aeruginosa*, the signaling mechanism regulates the gene expression of superoxide dismutase and catalase enzymes, which mediate hydrogen peroxide resistance [64]. Deficiency in quorum sensing is linked with lower EPS production, resulting in thinner and less dense biofilm formation. Such mutant or deficient biofilms are susceptible and vulnerable to antibiotics. As a result, researchers are developing a new field of study called “quorum quenching”, which aims to identify compounds with quorum-quenching capabilities.

### 3.4. Increased Genetic Exchange

Biofilms facilitate horizontal gene transfer (HGT), allowing for the exchange of genetic material between bacteria, particularly genes that confer resistance to antibiotics through the transfer of plasmids via conjugation. This process can accelerate the spread of resistance within the biofilm community. The introduction of genetic diversity leads to the adaptation, evolution, and survival of bacteria in adverse environments. Plasmid transfer is more efficient in biofilms compared to planktonic cells due to the proximity of bacterial cells in biofilms. Furthermore, certain bacteria can extract DNA from the biofilm matrix. The hydrated matrix creates ideal conditions for transformation in bacteria [65]. Antibiotic resistance gene sequences are over 100 times more common in biofilms than in planktonic cells [66].

### 3.5. Persister Cells

Persister cells are a subpopulation of biofilms that are genetically similar to active cells but are more tolerant and resistant to antibiotics due to the differences in physiological states [67]. Studies on ATP-dependent persister formation show that lower ATP levels can reduce the efficiency and accuracy of antibiotic-targeted cellular activities. Consequently, the antibiotic’s potential to target and disrupt crucial cellular operations is diminished [68]. In endocarditis, *S. aureus* develops biofilms on the heart valve. The bacteria’s altered metabolism within the biofilm makes the infection difficult to eliminate, often requiring prolonged or high-dose antibiotic therapy or even surgical intervention [44]. *M. tuberculosis* infects the lungs and forms biofilm-like structures. The presence of persister cells within these structures contributes to the chronic nature of the disease and the need for prolonged, multidrug antibiotic regimens.

### 3.6. Adaptive Changes in Gene Expression

Stress response in the immobilized cells inside the biofilm matrix can alter the gene expression, potentially increasing resistance to the action of biocides [44]. The multiple antibiotic resistance (mar) operons (marRAB) regulate the expression of many genes in *E. coli* and support the multidrug-resistant phenotype. MarR serves as a transcriptional auto-repressor of the marRAB operon, suppressing its expression, while MarA functions as an auto-activator of the marRAB operon [69]. MarA also reduces antibiotic entry and quinolone-induced DNA damage by upregulating genes necessary for lipid trafficking [70]. Most bacteria are fermentative, producing enzymes for the repair and breakdown of oxidizing agents, and depict the oxidizing stress response. After exposure to sub-inhibitory levels within a few hours, these stress-responding cells become more resistant to the damaging factors. In *E. coli*, several defense genes encoding DNA repair enzymes and catalysts such as superoxide dismutase, hydroperoxide reductase, and alkyl glutathione reductase have been identified [44]. Additionally, several regulatory genes such as oxyR and soxR have been identified, which control intracellular redox potential and generate a stress response when the cell is exposed to oxidizing agents. Compared to planktonic cells, biofilm-forming cells undergo mutation at a higher rate, potentially leading to increased antibiotic resistance. In biofilms, the accumulation of eDNA can acidify the surrounding environment, and this acidic pH promotes the *P. aeruginosa* antibiotic resistance phenotype. This leads to the aminoarabinose modification of Lipid A and the production of spermidine on the bacterial outer cell membrane. These modifications reduce the entry of aminoglycosides and are necessary to prevent antimicrobial peptide binding, membrane damage, and killing [71].

### 3.7. Efflux Pumps

Efflux pumps are membrane proteins found in all types of bacteria. They are involved in exporting harmful toxic compounds like antibiotics, dyes, toxins, detergents, and waste molecules from bacterial cells into the surrounding environment. The genes for efflux pumps are located either in chromosomal DNA or in the plasmid [72]. The efflux system enables bacteria to persist in harsh environments, including those with antimicrobial compounds. Efflux pumps exhibit resistance to several antibacterial agents belonging to the same or different families by using energy to reduce the concentration of drugs in the cytoplasm to sub-toxic levels [73]. The overproduction of efflux pumps contributes to a multidrug resistance (MDR) phenotype, often in combination with other resistance mechanisms such as target modification and antibiotic inactivation [74]. Efflux pumps function as a regulatory mechanism, affecting the permeability of the cell membrane by actively eliminating hydrophilic molecules and controlling the production of porins, which in turn regulates the entry of hydrophilic solutes into bacterial cells, thereby decreasing the diffusion of lipophilic solutes [75,76]. The biofilm’s efflux pumps and antibiotic-degrading enzymes work together to cause treatment resistance and recurring infections, making these UTIs difficult to cure [58,77].

The combined effects of these factors pose a formidable challenge for treating bacterial infections associated with biofilm formation. Developing strategies to disrupt or penetrate biofilms is an active area of research aimed at improving the effectiveness of antimicrobial treatments.

## 4. Therapeutic Approaches for Controlling Biofilm-Related Infections

**Figure 4:**
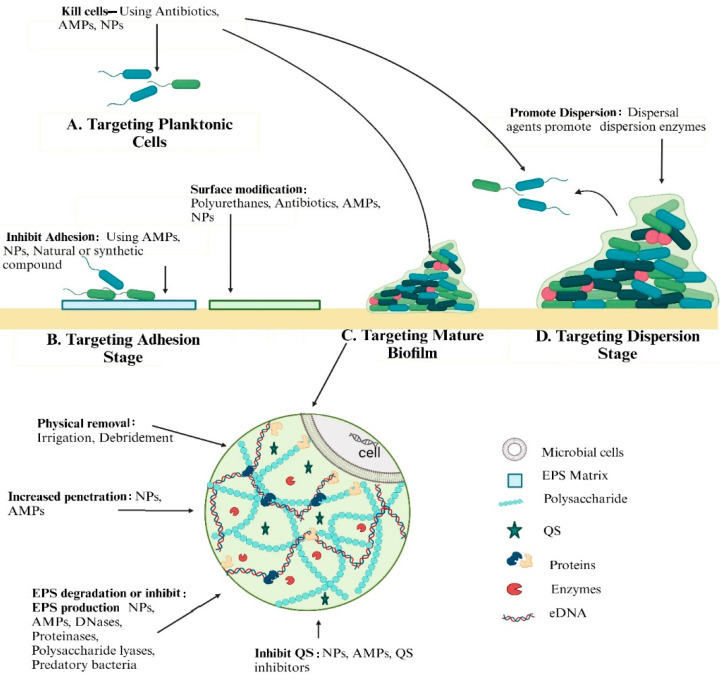
Strategies to eliminate biofilm at different growth stages (AMPs = Antimicrobial Peptides, NPs = Nanoparticles, QS = Quorum Sensing).

The presence of a protective extracellular matrix and persister cells within the biofilm, along with reduced cell growth and limited drug penetration, collectively hinder antimicrobial effects [4]. To combat this problem, several novel and unique approaches have been thoroughly examined (Figure 4).

Different methods are employed to eliminate biofilms at distinct stages of their growth (Table 4). Currently, there are three main approaches for controlling biofilm-related infections: (i) blocking the attachment and formation of biofilms, (ii) strategies targeting biofilm formation and maturation stage, or (iii) the elimination of biofilm chemically or mechanically [78,79].

### 4.1. Blocking the Attachment and Formation of Biofilms

The strategies to counter biofilm formation focuses on disrupting key stages, such as adhesion, maturation, EPS development, and dispersion. Surface modifications, nanomaterials, antimicrobial peptides, quorum-sensing inhibitors, and enzymatic approaches target initial adhesion and biofilm stabilization. Enzymatic disruption of the EPS matrix enhances biofilm removal, while dispersion strategies promote planktonic release for easier elimination. These methods offer innovative solutions to combat biofilm-associated infections in medical and industrial settings.

#### 4.1.1. Strategies for Targeting Initial Adhesion Stage

##### Anti-Biofilm Surface

Anti-biofilm surfaces are developed to prevent the initial attachment of microbes, disrupting biofilm formation and offering a proactive approach to inhibiting microbial colonization [80,81]. Materials such as certain polymers, metals, and nanoparticles can actively repel or kill microbial cells upon contact [82,83]. Polyethylene glycol (PEG) is a common polymer used to impart hydrophilic qualities. Surface coatings integrated with broad-spectrum antibiotics are utilized to inhibit biofilm growth on medical devices [84]. These devices have been extensively utilized in clinical trials, particularly in ICU settings [85,86]. Antimicrobial catheters have been demonstrated to improve outcomes even in the presence of bacteremia [87]. Hydrogels possess strong functional group density, biocompatibility, and lubricity; these qualities have led to their application in coating medical equipment. Additionally, the design of surfaces with biomimetic properties inspired by natural systems has shown promise in anti-biofilm strategies [88]. By mimicking surfaces found in nature, such as the lotus leaf, which repels water and contaminants, researchers have developed surfaces that deter microbial adhesion [89]. Some researchers have proposed an approach to disrupt adhesion by using chemicals that mimic bacterial adhesin receptors and induce anti-adhesion effects by preventing bacteria from attaching to the host [90]. α-mannosides function as an analog of bacterial adhesin receptors that interfere with FimH1 in catheter-related urinary tract infections (CAUTIs). This treatment scheme disrupts the adhesion process, preventing bacterial colonization [91]. Photodynamic therapy (PDT) has emerged as an innovative strategy for generating anti-biofilm surfaces [92]. By incorporating photosensitizers into surface coatings, exposure to light activates the photosensitizers, generating reactive oxygen species that can damage and kill attached microbial cells. This dynamic approach not only prevents biofilm formation but also provides a mechanism for actively disrupting established biofilms on surfaces.

#### 4.1.2. Strategies Targeting Biofilm Formation and Maturation Stage

##### Nanomaterial-Based Approaches

Nanomaterial-based treatments hold great promise in the fight against hard-to-treat bacterial infections. Due to their distinct size and physical characteristics, nanoparticles can target biofilms and lead to their eradication. Table 5 shows important classes of nanomaterials for antimicrobial applications [93]. Research indicates that disabling quorum-sensing molecules with different types of nanoparticles, such as liposome-based nanoparticles and metal-based nanoparticles, can prevent the production of biofilms and virulence factors.

##### Antimicrobial Peptides

Antimicrobial polypeptides are a diverse group of naturally occurring peptides, isolated from various kinds of living organisms, with an inherent broad range of antimicrobial properties against viruses, bacteria, fungi, and protozoa. They can be synthesized chemically. The human cathelicidin peptide LL-37 is one of the few AMPs that exhibits anti-biofilm activity. LL-37 is derived from human CAP-18 (18-kDa Cationic antimicrobial protein) protein (Table 6). These peptides can disrupt biofilms through different mechanisms. AMPs can target the bacterial membrane and break down the extracellular matrix, destabilizing the biofilm structure [94,95]. Moreover, AMPs may interfere with the initial stages of biofilm formation by preventing the adhesion of bacteria to surfaces and promoting cell dispersion in the early stages. This inhibitory effect can impede the development of mature biofilms [96]. Antimicrobial polypeptides may also work synergistically with other antimicrobial agents, such as nanoparticles (NPs) and antibiotics, enhancing their uptake and overall efficacy in combating biofilm-associated infections [97,98]. However, natural AMPs often exhibit proinflammatory effects and have poor stability, which limits their clinical application [99]. To overcome the toxicity and disadvantages associated with AMPs, low doses of AMPs are administered [80,99].

##### Quorum-Sensing Inhibitors

Interference can occur through various mechanisms by acting on different steps of QS signaling: (i) inhibition of the biosynthesis of signaling molecules; (ii) enzymatic degradation or modification of signaling molecules; (iii) blocking signal reception [79]. Mutation of the gene-producing signaling molecules is reported to be effective for inhibiting the synthesis of QS molecules. Quorum-quenching agents, such as bacterial-derived enzymes like lactonases and acylase extracts obtained from marine bacteria, as well as phytochemicals are used to prevent the accumulation of QS molecules [100]. These molecules are highly stable, show high efficacy, and have a broad spectrum of activity. Phytochemicals and crude extracts act as biofilm modulators by targeting the bacterial signal pathways. By disrupting quorum sensing, it becomes possible to interfere with the coordinated activities of bacterial populations, making them less virulent or hindering biofilm formation. This approach presents the potential for developing novel antimicrobial drugs or therapies that aim to attenuate bacterial pathogenicity without directly killing bacteria. Quorum-sensing inhibitors (QSIs) such as baicalin, cinnamaldehyde, hamamelitannin, N-(2-pyrimidyl) butanamide (C11), and furanone C-30 are used in combination with antibiotics. Researchers are exploring the potential of quorum-quenching agents to combat bacterial infections, enhance the efficacy of antibiotics, and mitigate the impact of bacterial biofilms in various settings [101]. However, gaps exist in understanding how these treatments can be optimized and applied effectively in practical situations.

#### 4.1.3. Strategies for Targeting EPS Through Enzymatic Disruption

Biofilm dispersal is a spontaneous process initiated in response to adverse environmental cues, such as nutrient deficiency, a decline in oxygen availability, or an increase in the concentration of nitric oxide (NO) [102]. The EPS matrix contains diverse types of proteins, glycoproteins, glycolipids, ions, and nucleic acid (e-DNA) that provide protection and support to bacterial cells [103]. Targeting these components with degrading enzymes is useful in controlling biofilm. Three groups of degrading enzymes have been identified: polysaccharide-degrading hydrolases that act on EPS, nucleases that target eDNA, and proteases that target proteins [58]. Dispersin B, alignate lyase (AlgL), and α-amylase are examples of glycoside hydrolyses. They are used in combination with other antimicrobial agents to increase their efficiency. Bovine deoxyribonuclease I (DNase I) has the potential to digest eDNA. DNase I disperses biofilms of several bacterial species without affecting bacterial viability [104]. Additionally, a DNase-mimetic artificial enzyme (DMAE) has been developed for anti-biofilm applications, showcasing strong cleavage ability, good operational stability, the ability to suppress biofilms for extended periods, and excellent dispersal activity of biofilms [58]. The peptide bond can be hydrolyzed by proteases, resulting in biofilm dispersal as they cleave proteins that are essential to maintain the biofilm matrix’s physical integrity [105]. Due to their capacity to eliminate biofilm, serine proteases such as trypsin and proteinase K have been extensively researched. Furthermore, the cell wall-associated protein EbpS can be cleaved by a set of six staphylococcal serine proteases (Spl operons), which are involved in *S. aureus* biofilm dispersal [106].

#### 4.1.4. Strategies of Targeting Dispersion Stage

The last stage of the biofilm life cycle, known as the dispersal phase, is a distinct phase representing the transition from biofilm to free planktonic bacteria, which are easier to eliminate. As a result, the active dispersal of biofilms has been investigated in several studies as a potential strategy for controlling biofilms. However, it is crucial to carefully manage the dispersion stage to prevent unintended colonization in undesirable areas.

After reaching a certain size, bacteria actively disperse by disrupting the EPS of the biofilm. One of the primary substances released by bacteria during the biofilm dispersal stage is D-type amino acids [107]. Consequently, therapeutic approaches combining D-type amino acids with drugs have been suggested. Initially, the peptidoglycan of bacteria was labeled with a portion of the D-type amino acids to achieve efficient targeting. Additionally, EPS in biofilms can be effectively cleaved by some D-type amino acids. Interestingly, the cleavage effect is focused on bacterial biofilms, leaving normal cells unaffected. Several combination therapies have been developed using antibiotics and nanoparticles in combination with D-amino acids to effectively eradicate drug-resistant bacteria in biofilms [108].

### 4.2. Restricting Biofilm Growth

Restricting biofilm growth is a critical focus in combating biofilm-associated infections. Strategies like bacteriophage therapy exploit highly specific viruses to target bacteria [109], while combination therapies integrate antibiotics, enzymes, or nanoparticles to weaken biofilm defenses [110,111]. Modulating host immune responses further enhances biofilm clearance by improving immune recognition and balancing inflammation.

#### 4.2.1. Bacteriophage Therapy

Bacteriophages are biological entities that have intricate and coevolving connections with bacteria. They are highly specific to specific strains of bacteria. Phage therapy is a technique that uses bacteriophages for the treatment of pathogenic bacterial infections [112]. Bacteriophages are found to be more effective than antibiotics as they can easily penetrate the EPS. Phage cocktails leverage multiple phages to enhance bacterial lysis, extend host range, and mitigate resistance more effectively than single-phage treatments [113]. Synergistic combinations of phages with antibiotics amplify bacterial eradication and biofilm disruption. Genetically engineered phages improve specificity, biofilm degradation, and treatment efficacy. Enzymes derived from phages, such as depolymerases and lysins, dismantle biofilm matrices and potentiate antibiotic action. Additionally, the integration of phages with nanotechnology, disinfectants, or photodynamic techniques offers advanced approaches to tackle multidrug-resistant biofilm infections [109]. Bacteriophages attach to the bacterial membrane and insert their viral genetic material into the bacteria. The virus uses the host machinery to produce new virions and interferes with the function of bacteria, halting its growth and bacterial infection. The infection-causing bacterial cell cannot proliferate; instead, it generates more phages [114]. Only virulent phages that can perform lytic cycles are significant for therapeutic use.

#### 4.2.2. Combination Therapies

Combination therapies enhance the potential for eradicating biofilms by targeting multiple aspects of their formation, stability, and resistance mechanisms [110]. One facet of combination therapy involves the simultaneous use of antibiotics with anti-biofilm agents [81,111]. Anti-biofilm agents, such as enzymes targeting the extracellular matrix or quorum-sensing inhibitors that disrupt microbial communication, complement antibiotics by weakening biofilm structures and rendering bacteria more susceptible to antimicrobial agents [115,116]. Enzymes like DNase and dispersin B can degrade the extracellular DNA (eDNA) and polysaccharides constituting the biofilm matrix, facilitating the penetration of antibiotics and other antimicrobial agents [88,117,118]. Photodynamic therapy (PDT) is another modality often combined with traditional antimicrobials [84,119]. The combination of PDT with antibiotics or other anti-biofilm agents enhances the overall efficacy, providing a multifaceted attack on biofilm communities [120]. Moreover, nanoparticles because of their unique physicochemical properties can disrupt biofilm structures, enhancing the overall efficacy of combination therapies [121].

#### 4.2.3. Host Immune Modulation

Biofilms possess the ability to evade immune surveillance and exhibit increased resistance to host defenses. The modulation of the host immune response represents a promising strategy to bolster the clearance of biofilm infections by enhancing the immune system’s ability to recognize and eliminate biofilm-embedded pathogens [117,118]. Immunomodulatory agents, including cytokines and Toll-like receptor agonists, have been explored to stimulate immune cell responses and improve the recognition and engulfment of biofilm-associated microbes [122,123,124]. In addition to enhancing phagocytosis, efforts have been directed towards modulating the inflammatory response to balance immune activation and tissue damage. Chronic inflammation associated with biofilm infections can contribute to tissue destruction and further protect biofilm-embedded bacteria [125,126]. Immunomodulators, such as anti-inflammatory cytokines or resolution-phase mediators, are designed to modulate the inflammatory response by fostering a controlled and effective immune response without causing excessive tissue damage [127,128].

Furthermore, the development of biofilm-degrading enzymes and antibodies that specifically target biofilm matrix components represents a promising avenue for enhancing host immune responses [129]. These agents can act in synergy with the immune system by disrupting the protective matrix, rendering biofilm-encased microbes more vulnerable to immune attack [130]. These strategies hold significant potential for overcoming the challenges posed by biofilm-associated infections, providing new avenues for therapeutic interventions in clinical settings.

### 4.3. Elimination of Biofilm Physically/Mechanically

Irrigation and debridement are commonly used medical procedures for physically removing or cleaning a wound or a body cavity. Irrigation is the process of washing with a sterile saline or antiseptic solution over the affected area to remove bacteria, debris, and biofilm components from the area [131]. Debridement involves removing the bacterial biofilm and damaged or infected tissue from a wound or afflicted area. This can be accomplished in several ways, such as via enzymatic debridement agents, mechanical approaches, or surgical removal [131]. Irrigation and debridement followed by vigorous antimicrobial treatment have been extensively employed for oral, wound, and prosthetic joint biofilm-related infections; however, it has also been discovered that biofilms spread across surfaces and contribute to the persistence of bacteria even after the administration of these treatments [124]. Certain novel technologies, such as cavitating jets and low-intensity intermittent ultrasonication-induced microbubble bursting have been employed in combination with these conventional treatments.

## 5. System Biology and Advanced Molecular Techniques

Integrating advanced molecular techniques and systems biology approaches represents a cutting-edge strategy aimed at unraveling the intricate dynamics of biofilm resistance mechanisms. Traditional methods often fall short in capturing the complex interactions occurring within biofilms, necessitating a more holistic and sophisticated approach to dissect the molecular intricacies of biofilm-mediated antibiotic resistance. Advanced molecular techniques encompass a broad array of methodologies designed to probe the molecular and genetic landscape of biofilm communities [124]. These include high-throughput sequencing technologies, such as RNA sequencing (RNA-Seq) and metagenomics, which allow for the comprehensive profiling of gene expression and microbial community composition within biofilms [125,126]. Techniques such as confocal laser scanning and super-resolution microscopy enable the visualization of the biofilm architecture at unprecedented resolutions [127,132]. This allows researchers to observe the spatial organization of microbial cells and the extracellular matrix, providing crucial insights into the structural basis of biofilm resistance. Live-cell imaging facilitates the real-time monitoring of biofilm behavior and response to antimicrobial treatments [128]. In parallel, systems biology approaches leverage computational and mathematical modeling to integrate large-scale omics data and generate predictive models of biofilm behavior [129]. Systems biology enables the construction of intricate networks representing the interactions between genes, proteins, and metabolites within biofilms. This holistic view aids in identifying key nodes of biofilm resistance and predicting potential targets for therapeutic intervention.

## 6. Conclusions

This review examines the challenges of bacterial biofilm-associated infections, focusing on antibiotic resistance mechanisms. It highlights the need for innovative therapies to overcome the limitations of current antimicrobials. By exploring genetic and phenotypic biofilm adaptations, potential vulnerabilities can be targeted. Innovative approaches like nanomaterials, quorum-sensing inhibitors, bacteriophages, and combination therapies are emphasized to improve treatment efficacy. The integration of diverse expertise and technologies is crucial for advancing understanding and developing effective solutions. Continued research is vital to address biofilm-associated infections and improve clinical outcomes.

## Figures and Tables

**Table 1:** Molecule/components and their role in biofilm formation.

Molecule/Component	Type	Role in Biofilm Formation
Polysaccharides (e.g., Polysaccharide Intercellular Adhesin)	Extracellular Polymeric Substances	A major component of the biofilm matrix, providing structural support and protection.
Proteins (e.g., adhesins)	Extracellular Polymeric Substances	Facilitate bacterial adhesion to surfaces and contribute towards biofilm stability.
Autoinducers (AI)	Quorum-Sensing Molecules	Regulates gene expressions associated with biofilm formation based on cell density. Examples: AI-2.
c-di-GMP	Signaling Molecule	Acts as a secondary messenger to promote biofilm formation by enhancing EPS production and bacterial adhesion.
cAMP	Signaling Molecules	Modulates biofilm formation and bacterial motility by influencing gene expression.
Fimbriae and Pili	Surface Structure	Help bacteria adhere to surfaces and initiate biofilm formation.
Flagella	Surface Structures	Assist in initial attachment of bacteria to surfaces and motility towards biofilm formation sites.

**Table 2:** Key quorum-sensing molecules and their roles in biofilm regulation.

Bacterial Species	Quorum-Sensing Molecule	Role in Biofilm Formation
*P. aeruginosa* (PAO1 strain)	N-acyl homoserine lactones (AHLs)	Regulates release of extracellular DNA (eDNA) and biofilm structure [43]
*P. aeruginosa* (PA14 strain)	N-acyl homoserine lactones (AHLs)	Governs synthesis of PEL Exopolysaccharide [43]
*S. aureus*	Autoinducing Peptide (AIP)	Orchestrates biofilm dispersion [44]
*Bacillus subtilis*	Surfactin	Promotes biofilm formation by inducing extracellular matrix production [45,46]

**Table 3:** Regulatory enzymes and the functional role of c-di-GMP in various bacterial species.

Bacterial Species	Regulatory Enzyme/Protein	Function of c-di-GMP
*P. fluorescens*	LapD (effector protein)	Binds c-di-GMP to promote LapA transportation to the cell surface. Initiates biofilm formation [49,50]
*Escherichia coli*	YcgR (effector protein)	Integrates c-di-GMP levels with flagellar motor components. Regulates transition from motile to sessile lifestyle (high c-di-GMP levels favor sessile states, while low levels support motility) [48,51]
*Vibrio cholerae*	VieS/VieA sensor kinase/response regulator pair	VieA PDE degrades c-di-GMP when phosphorylated by VieS. Reduced c-di-GMP levels repress biofilm formation and vps gene expression [52]

**Table 4:** Therapeutic approaches utilized to remove biofilms.

Stage of Biofilm	Characteristics	Target	Therapeutic Approaches
Initial Adhesion	Initial adhesion is reversible in nature and later becomes irreversible.	Adhesion proteins like adhesins and surface.	Surface modification
Early Formation	EPS formation. Active intercellular Communication by QS.	QS molecules; Polysaccharide Intracellular Adhesin (PIA); eDNA; EPS.	Nanoparticles, Antimicrobial peptide
Maturation	Mature EPS. Chemical gradient and hypoxic microenvironment present. Changes in the metabolism of bacteria. Decrease in pH.	Persister cells and dormant bacteria. EPS degradation or inhibition of EPS production.	Nanoparticles, Antimicrobial peptides, QS inhibitors, etc.
Dispersion	Accumulation of biofilm residues, secretion of enzymes like D-amino acids, and other substances to destroy EPS.	Planktonic cells produced by disruption of biofilm.	Dispersal enzymes

**Table 5:** Applications of some selected nanomaterials in antimicrobial therapies.

Nanomaterial Type	Properties/Mechanisms	Application
Metal-based Nanoparticles	Toxicity via ROS production/membrane disruption	Silver-based NPs are effective against multidrug-resistant bacterial infections.
Carbon-based Nanomaterials	Bactericidal action through membrane damage and by blocking adhesion	Prevent biofilm formation
Polymeric Nanomaterials	Synthetic polymers resemble and imitate antimicrobial peptides	Serve as nanocarriers to enhance drug solubility and stability. Inhibits biofilm formation
Nanocomposites	Hybrids of inorganic and organic nanoparticles showing synergistic antimicrobial activity	Enhanced efficacy against bacteria
Nano-emulsions	Nanosized emulsions and encapsulation of bioactive compounds	Used to improve the solubility and stability of essential oils and medicines
Liposomes	Vesicles with phospholipid bilayers and aqueous core. Drug delivery vehicles encapsulate hydrophilic and hydrophobic drugs in their core and phospholipid membrane, respectively	Targeted and effective drug delivery vehicles. Effective against microbes
Smart Nanomaterials	Respond to stimuli (pH, toxins, light, temperature, ultrasound)	Targeted antimicrobial action

**Table 6:** Selected polypeptides and their functions as antimicrobials.

Type of Antimicrobial Proteins	Functions
Cathelicidins	Have broad-spectrum antimicrobial activity and can modulate immune responses.
Defensins	Disrupt microbial cell membranes which leads to cell death.
Lactoferrin	Sequesters iron, an important nutrient required for the growth of bacteria, and has direct antimicrobial activity.
Histatins	Exhibit antifungal activity, especially against *Candida* species.
Thrombocidins	Have antimicrobial activity against bacteria and fungi and can modulate inflammation.
Perforin	Forms pores in the cell membrane and induce apoptosis.
